# Tofacitinib and Baricitinib Are Taken up by Different Uptake Mechanisms Determining the Efficacy of Both Drugs in RA

**DOI:** 10.3390/ijms21186632

**Published:** 2020-09-10

**Authors:** Jan Amrhein, Susanne Drynda, Lukas Schlatt, Uwe Karst, Christoph H. Lohmann, Giuliano Ciarimboli, Jessica Bertrand

**Affiliations:** 1 Experimental Nephrology, Department of Internal Medicine D, University Hospital Münster, 48149 Münster, Germany; jan.amrhein@web.de (J.A.); gciari@uni-muenster.de (G.C.); 2 Department of Orthopedic Surgery, Otto-von-Guericke University, 39120 Magdeburg, Germany; susanne.drynda@med.ovgu.de (S.D.); christoph.lohmann@med.ovgu.de (C.H.L.); 3 Institute of Inorganic and Analytical Chemistry, University of Münster, 48149 Münster, Germany; lukas.schlatt@uni-muenster.de (L.S.); uk@uni-muenster.de (U.K.)

**Keywords:** RA, Tofacitinib, Baricitinib, organic cation transporter, MATE1

## Abstract

Background: Rheumatoid arthritis (RA) is a systemic autoimmune disease in which synovial fibroblasts (SF) play a key role. Baricitinib and Tofacitinib both act intracellularly, blocking the ATP-binding side of JAK proteins and thereby the downstream signalling pathway via STAT-3. Therefore, we investigated the role of organic cation transporters (OCTs) in Baricitinib and Tofacitinib cellular transport. Methods: OCT expression was analysed in SF isolated from RA and osteoarthritis (OA) patients, as well as peripheral blood mononuclear cells. The interaction of Baricitinib and Tofacitinib with OCTs was investigated using quenching experiments. The intracellular accumulation of both drugs was quantified using LC/MS. Target inhibition for both drugs was tested using Western blot for phosphorylated JAK1 and STAT3 upon stimulation with IL-6. Results: MATE-1 expression increased in OASF compared to RASF. The other OCTs were not differentially expressed. The transport of Baricitinib was not OCT dependent. Tofacitinib; however, was exported from RASF in a MATE-1 dependent way. Tofacitinib and Baricitinib showed comparable inhibition of downstream signalling pathways. Conclusion: We observed different cellular uptake strategies for Baricitinib and Tofacitinib. Tofacitinib was exported out of healthy cells due to the increased expression of MATE1. This might make Tofacitinib the favourable drug.

## 1. Introduction

Rheumatoid arthritis (RA) is a systemic autoimmune disease that predominantly affects synovial joints, causing progressive polyarthritis, joint destruction and disability [1]. Synovial fibroblasts (SF) are key players in the development of RA [2]. In healthy synovial joints the synovium is formed of a few fibroblast layers, which mainly regulate the production of synovial fluid [3]. Fibroblasts also play a role in the inert immune system, carrying Toll-like receptors and being able to secrete cytokines [4,5]. In RA, fibroblasts evolve a tumour-like phenotype which transforms them to Rheumatoid Arthritis synovial fibroblasts (RASF) [6]. RASF acquire an aggressive phenotype with increased proliferation, loss of cell–cell contacts and joint invasiveness, where they secrete proinflammatory cytokines and interact with other immune and stroma cells to perpetuate the inflammatory reaction [2].

Cytokine signalling is a crucial driver of inflammation processes in RA. Besides tumour necrosis factor alpha (TNF-α), Interleukin-6 (IL-6) mediates major inflammatory signalling pathways in RA [7]. Among other cytokines, it predominantly affects the JAK/STAT signalling. Janus kinases (JAKs) are non-receptor protein tyrosine kinases that consist of JAK1, JAK2, JAK3 and TYK2. IL-6 binds to the IL-6Rα/gp130 complex that is linked to JAKs [8]. Upon binding of IL-6 to the receptor complex JAKs get phosphorylated by each other [9]. The cytosolic signal transducer and activator of transcription proteins (STATs) are able to bind to phosphorylated residues of the JAKs and get phosphorylated as well. They dimerize, translocate into the cell nucleus and act as transcription factors for the production of further proinflammatory cytokines, as well as cell differentiation and cell proliferation inducing factors [8].

Baricitinib and Tofacitinib are both relative new drugs, so called tyrosine kinase inhibitors (TKIs), approved and recommended by the European League Against Rheumatism (EULAR) for the treatment of RA [10,11]. These TKIs both act intracellularly, where they block the ATP-binding side of JAK proteins and thereby the downstream signalling pathway via STAT proteins. The binding of JAK inhibitors reduces cell differentiation, proliferation and production of proinflammatory cytokines [12,13]. Patients, that show an inadequate response to the disease-modifying drugs methotrexate (MTX) and/or biologics, may receive Tofacitinib or Baricitinib. Therefore, it is important to determine the optimal treatment options for these patients with regard to efficacy and safety of Tofacitinib and Baricitinib. To date, it is not known why different TKIs exert different effects in patients with the same disease. Differences in the uptake mechanism could explain these differences.

Around 40% of all orally administered drugs show cationic characteristics, and therefore need specific transport systems to penetrate nonpolar cell membranes to reach their intracellular target [14]. Previous studies showed that other TKIs like Imatinib and Saracatinib were dependent on polyspecific (meaning that they can accept structurally different substances as substrate) organic cation transporters (OCTs) to reach their intracellular target [15,16,17]. OCTs are part of the solute carrier (SLC) family [18]. This family includes the human organic cation transporters (hOCT1, hOCT2, hOCT3), the novel organic cation transporters (hOCTN1, hOCTN2), and the multidrug and toxin extrusion proteins (hMATE1, hMATE2k). Many of these OCTs share the same substrates, but every transporter has an individual substrate/inhibitor interaction profile. Whereas hMATE1, hMATE2k and hOCTN1 mediate a H+/organic cation (OC) antiport, hOCT1, hOCT2 and hOCT3 transport along the electrochemical gradient of their substrates. OCTs are widely expressed in different cells and are essential for the secretion of organic cations (OCs) in the liver and kidney [19]. Even though they transport mainly endogenous and exogenous OCs, interactions with zwitterions and anions have been reported [20,21]. 

As Tofacitinib and Baricitinib are established drugs for the treatment of RA, we investigated the uptake pathways of these drugs under RA relevant conditions focussing on their interaction with different OCTs and on their therapeutic efficiency in RASF.

## 2. Results

### 2.1. Tofacitinib Could Be a Target for OCT Mediated Cellular Uptake

**Figure 1:**
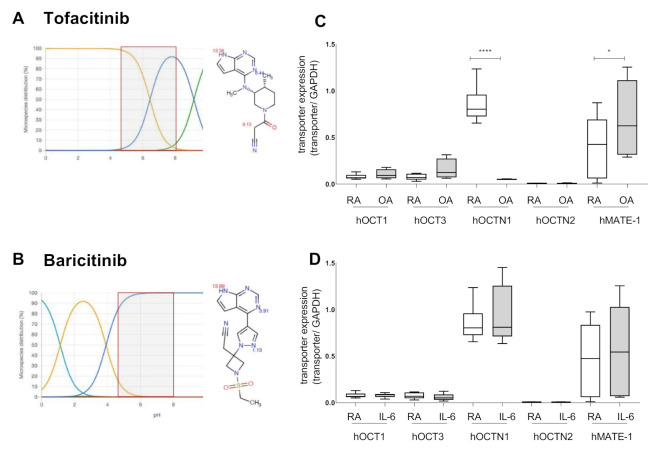
Tofacitinib could be a target for OCT mediated cellular uptake. (**A**,**B**) pKA prediction for Tofacitinib (**A**) and Baricitinib (**B**). The yellow curves indicate the presence of cationic form, the blue curves indicate the neutral form and the green curves of the anionic form of the substances at the given pH-values. The red boxes indicate the physiological pH-range. (**C**) Quantitative RT-PCR for the expression pattern of OCTs, and *hMATE-1* from OASF and RASF. GAPDH was used as housekeeping gene for normalization. (**D**) Quantitative RT-PCR investigating the expression pattern of OCTs, and *hMATE-1* in RASF after stimulation with 10 ng/mL IL-6 for 24 h. *GAPDH* was used as housekeeping gene for normalization. Statistical analyses were performed using an ordinary one-way ANOVA and Sidak correction for multiple testing. *: *p* < 0.05; ****: *p* < 0.0001

Predictions on the pKa of Baricitinib and Tofacitinib, using the online tool Chemicalize of ChemAxon, showed that Baricitinib is not charged in neutral pH solutions (red box Figure 1B), whereas Tofacitinib is partially positively charged (red box, Figure 1A). Adjusting the pH to more acidic values, like in RA synovial fluid, supposes an increase of positively charged species of the two drugs. However, Baricitinib would largely stay uncharged, whereas Tofacitinib gets more positively charged making it a potential substrate for organic cation transporters. Therefore, we investigated the expression pattern of OCTs in OASF and RASF (Figure 1C). We observed no difference in the expression of *hOCT1* and *hOCT3* between RA and OA synovial fibroblasts. *hOCT2* was not detectable. *hOCTN2* was also only weakly expressed and no difference was seen between RASF and OASF. RASF, however, expressed significantly more *hOCTN1* (F (9, 44) = 12.06, 95%CI: 0.4480 to 1.157, *p* < 0.0001). *MATE-1* was lower expressed in RASFs compared to OASF (F (9, 44) = 12.06, 95%CI: −0.6278 to −0.01494, *p* = 0.0358). This effect is even more pronounced in PBMCs from RA and OA patients (supplementary Figure S1). As both TKIs influence the IL-6 dependent pathways, we stimulated RASF with IL-6 and investigated the changes in the OCT expression pattern (Figure 1D). We observed an increase of *hMATE-1* expression, levelling out the differences between RASF and OASF. The other analysed OCTs were not changed in their expression.

### 2.2. Baricitinib Uptake Is Not Transporter Dependent

**Figure 2:**
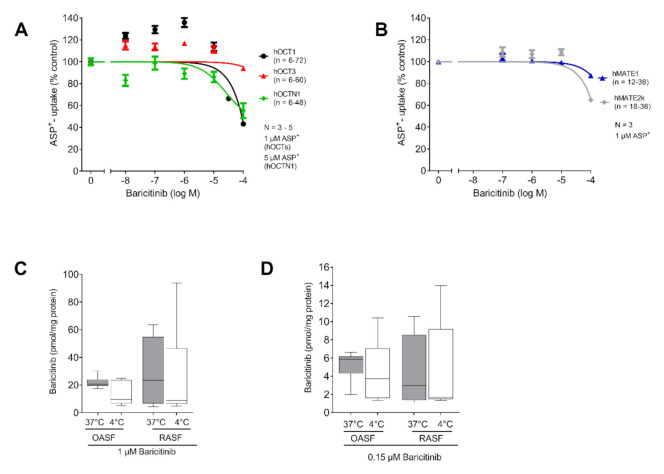
Baricitinib uptake is not transporter dependent. (**A**) ASP^+^ determined apparent affinity of Baricitinib to hOCT1, hOCT3 and hOCTN1. No significant interaction was observed (*n* = 3–5). (**B**) ASP^+^ determined affinity of hMATE1 and hMATE2k after intracellular acidification (*n* = 3). No significant interaction was observed. Data in (**A**,**B**) are presented as mean ± SEM and fitted using a non-linear fit. (**C**) LC/MS measurement of temperature dependent Baricitinib (1 µM) uptake in RASF and OASF (F (1.332, 10.21) = 1.570; *p* = 0.2470; *n* = 3). (**D**) LC/MS measurement of temperature dependent Baricitinib uptake (0.15 µM) in RASF and OASF (F (2.071, 16.56) = 0.44, *p* = 0.6578, *n* = 3). Statistical analyses were performed using a RM ANOVA. No post-hoc testing was performed, as the ANOVA was not significant. Data in (**C**,**D**) are presented as box plot with whiskers indicating the min and max values, as well as the median.

First, we investigated a potential interaction of Baricitinib with different OCTs using the ASP^+^ quenching method as a readout. We observed no significant interaction with any of the expressed OCTs in a physiological range of Baricitinib concentration (Figure 2A,B). To investigate a potential transporter dependent accumulation of Baricitinib in SF, we used OASF and RASF for LC/MS determination of intracellular Baricitinib concentrations. We observed no change in Baricitinib concentration neither depending on the temperature, nor depending on the disease (RASF vs OASF). We observed a higher intracellular Baricitinib concentration using 1 µM (Figure 2C), compared to the approximate serum concentration of 0.15 µM Baricitinib (Figure 2D). However, no temperature-dependent change in Baricitinib concentration was detected, indicating that the uncharged Baricitinib might be able to penetrate the cell membrane without active transport.

### 2.3. MATE-1 Mediates Tofacitinib Transport

**Figure 3:**
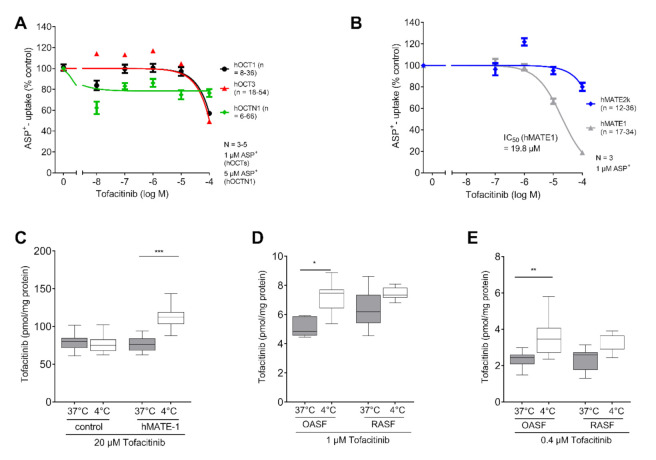
MATE-1 mediates Tofacitinib transport. (**A**) ASP^+^ determined apparent affinity of Tofacitinib to hOCT1, hOCT3 and hOCTN1. No significant interaction was observed (*n* = 3–5). (**B**) ASP^+^ determined apparent affinity of hMATE1 and hMATE2k after intracellular acidification (*n* = 3). MATE-1 shows an apparent affinity IC50 = 19.8 µM to MATE-1. Data in (**A**,**B**) are presented as mean ± SEM and fitted using a non-linear fit. (**C**) LC/MS determined intracellular Tofacitinib concentration of HEK cells (control) and HEK cells overexpressing MATE-1 at 37 °C and 4 °C (*n* = 3). (**D**) LC/MS measurement of temperature dependent Baricitinib (1 µM) uptake in RASF and OASF (*n* = 3). (**E**) LC/MS measurement of temperature dependent Baricitinib uptake (0.4 µM) in RASF and OASF (*n* = 3). Statistical analyses were performed using a RM ANOVA and Sidak correction for multiple testing. Data in (**C**,**D**) are presented as box plot with whiskers indicating the min and max values, as well as the median. *: *p* < 0.05; **: *p* < 0.01; ***: *p* < 0.005

To test the affinity of Tofacitinib to different OCTs, we again performed the ASP^+^ quenching assay. We observed no interaction of Tofacitinib with the OCTs (Figure 3A). Next, we investigated the interaction with MATE transporters using previous acidification. Interestingly, we found concentration-dependent inhibition of ASP^+^ uptake by Tofacitinib in hMATE1 transfected HEK cells with an IC50 of 19.8 µM (Figure 3B). To validate this finding, we used hMATE1-transfected HEK cells and observed a significant decrease in Tofacitinib intracellular accumulation at 37 °C (F (1.805, 12.63) = 20.71, 95%CI: −39.45 to −23.47, *p* < 0.0001). This suggests a transporter mediated export of Tofacitinib via hMATE1 (Figure 3C). When investigating 1 µM Tofacitinib concentration in OASF at 37 °C and 4 °C, we again observed significantly lower Tofacitinib concentrations at 37 °C compared to 4 °C (1 µM) (F (2.123, 12.74) = 10.15, 95%CI: −3.405 to −0.9026, *p* = 0.0045) (Figure 3D). This effect was not as pronounced in RASF, which might be explained by the lower hMATE1 expression in RASF compared to OASF (F (2.123, 12.74) = 10.15, 95%CI: −2.549 to 0.6115, *p* = 0.225). Using the approximate serum concentration of Tofacitinib for therapeutic use, we found the same pattern again, indicating that this effect is also present at low Tofacitinib concentrations (OASF: F (1.969, 15.75) = 6.925, 95%CI: −2.118 to −0.3189, *p* = 0.012) (Figure 3E).

### 2.4. Tofacitinib and Baricitinib Showed Comparable Inhibition of IL-6-Induced STAT3-Phosphorylation 

**Figure 4:**
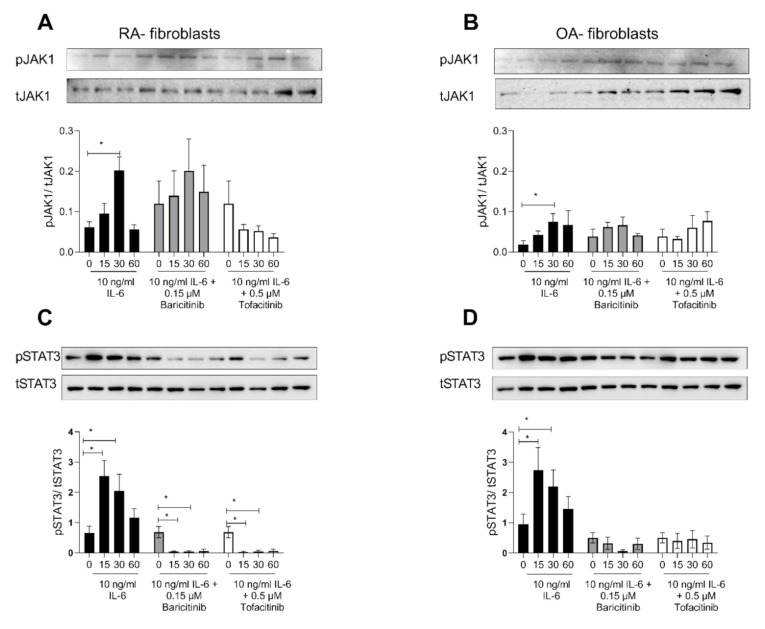
Tofacitinib and Baricitinib showed comparable inhibition of IL-6 induced STAT3- phosphorylation. (**A**) Western blot for pJAK1 compared to total Jak1 in RASF stimulated for 0, 15, 30 or 60 min with 10 ng/mL Il-6. Cells were also treated with 0.15 µM Baricitinib or 0.5 µM Tofacitinib. Quantification of at least 5 independent experiments are given in the graph underneath the blot. (**B**) Western blot for pJAK1 compared to total Jak1 in OASF stimulated with 10 ng/mL Il-6 and treated with 0.15 µM Baricitinib or 0.5 µM Tofacitinib. Quantification is given in the graph underneath the blot. (**C**) Western blot for pSTAT3 compared to total STAT3 in RASF stimulated for 0,15, 30 or 60 min with 10 ng/mL Il-6. Cells were also treated with 0.15 µM Baricitinib or 0.5 µM Tofacitinib. Quantification of at least five independent experiments is given in the graph underneath the blot. (**D**) Western blot for pSTAT3 compared to total STAT3 in OASF stimulated with 10 ng/mL Il-6 and treated with 0.15 µM Baricitinib or 0.5 µM Tofacitinib. Quantification is given in the graph underneath the blot. A two-way RM-ANOVA was performed for statistical testing. Data are presented as mean values with SEM. A *p*-value *p* ≤ 0.05 was considered to show statistical significance and is indicated by *.

As it was shown that Baricitinib and Tofacitinib are taken up by fibroblasts via different uptake mechanisms, we analysed the inhibition of JAK1 phosphorylation as well as the downstream target STAT3. Using 10 ng/mL IL-6 we activated the Jak1-STAT3 signalling pathway. We observed an increased time-dependent phosphorylation of Jak1 in RASF using IL-6 (untreated vs. 30 Min IL-6; 95%CI: −0.24 to −0.04; *p* = 0.02). Baricitinib did not influence phosphorylation JAK1 during the tested time course (Figure 4A). Tofacitinib, in contrast, time dependently inhibited the phosphorylation of JAk1 (Figure 4A). However, due to the very low amounts of pJAk1 detectable, these results did not reach statistical significance. OASF were less responsive towards IL-6 stimulation. They showed only a weak phosphorylation of Jak1 (untreated vs. 30 min IL−6 95% CI: −0.1 to 0.002; *p* = 0.05), and no difference was observed using Baricitinib or Tofacitinib to inhibit the IL-6 induced phosphorylation (Figure 4B). Next, we investigated the phosphorylation of STAT3. As expected, 10 ng/mL IL-6 resulted in an increased time dependent phosphorylation of STAT3 in RASF (Time effect: F(1.67, 33.37) = 8.62; *p* = 0.002). Using either Baricitinib or Tofacitinib, this phosphorylation was inhibited, indicating an efficient inhibition of IL-6 induced signalling using both TKIs (Treatment effect: F(2, 24) = 11.27; *p* = 0.0004). No significant difference was observed between both drugs (Figure 4C). We also investigated the efficacy of IL-6 blockade in OASF. There was less phosphorylation of STAT3 in unstimulated OASF, and these fibroblasts were also less responsive compared to RASF (Figure 4D). The treatment with either Tofacitinib or Baricitinib completely abolished the IL-6 induced phosphorylation of STAT3 (Treatment effect: F (1.179, 9.432) = 16.35; *p* = 0.002). Again, no difference was observed between both TKIs.

## 3. Discussion

Both tested TKIs, Tofacitinib and Baricitinib, are approved as therapeutic drugs for the treatment of RA. Tofacitinib predominantly inhibits JAK1 and JAK3, and to a lesser degree JAK2 [13]. Baricitinib mainly inhibits JAK2, and acts only to a minor degree on the phosphorylation of JAK1 and JAK3 [12].

Tofacitinib was the first JAK inhibitor approved by the FDA in 2012 and subsequently by the EMA in 2017 for use in patients with moderate-to-severe RA at a dose of 5 mg twice daily. Tofacitinib in combination with MTX is indicated for the treatment of moderate to severe active RA in adult patients who have responded inadequately to, or who are intolerant to, one or more disease-modifying antirheumatic drugs. Tofacitinib can be given as monotherapy in the case of intolerance to MTX, or when treatment with MTX is inappropriate. Baricitinib 2 mg once daily (as monotherapy or combination therapy) was approved for RA patients with inadequate response to one or more tumour necrosis factor antagonist therapies in the US and for csDMARD-IR in Canada, while Baricitinib 2 mg and 4 mg (as monotherapy or combination therapy) were approved for RA patients with csDMARD-IR in Europe. 

Variances in the clinical performance of both JAK inhibitors have been observed [22]. However, the reason for these differences are unknown given that they both TKIs target Janus Kinases resulting in a reduced STAT3 phosphorylation. However, each TKI has a different inhibitory profile against the different JAK isotypes [23,24]. This study aims to give an explanation for this observation by evaluating the intracellular uptake of both TKI into their targeted cells. A possible target in RA are synovial fibroblasts as they play an important role in the pathogenesis by contributing to joint destruction and producing cytokines [3]. RASF express several organic ion transporters which are capable of translocating TKIs, among them hOCTN1 and hMATE1, which have been previously reported to transport Saracatinib and Imatinib, respectively [14,15,16,17]. 

Baricitinib is not charged under physiological conditions, and therefore is no a target for organic cation transporters (Figure 1A). For this reason, we did not observe a transporter-mediated uptake in either the ASP^+^ quenching tests (Figure 1C), nor the LC/MS detection of temperature dependent Baricitinib accumulation (Figure 2). 

Investigating the role of organic cation transporters for the Tofacitinib accumulation in human RASF, we identified hMATE1 to predominantly mediate this transport (Figure 3C). Compared to OASF, hMATE1 expression is reduced in RASF (Figure 1C). We have previously shown that pro-inflammatory cytokines influence the MATE-1 expression [14]. RA is characterized by inflammatory processes that impact on various cellular activities [25]. Therefore, the influence of IL-6, which is also the main activator of JAK signalling, on OCT expression was analysed. IL-6 did not further impact on the expression of MATE-1 (Figure 1D). 

Tofacitinib is charged under physiological pH-conditions (Figure 1B). Because transport of organic cations mediated by MATE-1 is pH dependent, we observed an export of Tofacitinib [21]. The synovial fluid in RA patients has been reported to exhibit an acidic pH, under these conditions MATE-1 is expected to mediate efflux of Tofacitinib [25,26]. To investigate the intracellular concentration of both TKIs we chose the maximum plasma concentration for Tofacitinib and Baricitinib for our experiment. The concentration for Baricitinib was described as 150 nM, and 400 nM for Tofacitinib [27,28]. As expected, we did not observe a temperature-dependent increase of Baricitinib in fibroblasts (Figure 2D). This indicates that, due to its neutral charge, Baricitinib can penetrate the cell membrane without active transport. However, our data do not exclude that other transporters might contribute to the transport of Baricitinib into the cells. Tofacitinib in contrast is actively transported into fibroblasts. We found that MATE1 is the responsible transporter (Figure 3C). Interestingly, we found that OASF show a temperature-dependent lowering of Tofacitinib concentrations, indicating an active export of the drug from the cells (Figure 3D,E). This correlates with the increased MATE1 expression in OASF, which is reduced under inflammatory conditions. For this reason, in RASF, Tofacitinib is not exported. 

We investigated the potency of both TKIs in inhibiting IL-6-induced JAK1 phosphorylation. As expected, Baricitinib did not inhibit JAK1 phosphorylation in RASF (Figure 4A). OASF were less responsive to IL-6 treatment (Figure 4B,D). Investigating the downstream transcription factor for IL-6 signalling, we observed no difference between Baricitinib and Tofacitinib, indicating that both TKIs are efficiently inhibiting the inflammatory response (Figure 4C).

The results from this study indicate that Tofacitinib might be exported from healthy cells, thereby not inhibiting the JAK pathway. Under disease conditions; however, Tofacitinib stays in the diseased cells and effectively inhibits the disease pathway. We observed no difference in inhibition of IL-6-induced inflammatory signalling for Tofacitinib and Baricitinib. 

## 4. Conclusions

Thus, the differences in cellular uptake strategies for Baricitinib and Tofacitinib might explain the differences in clinical performance. Knowing that Tofacitinib is transported from healthy cells due to the increased expression of MATE1 might make it the more favourable drug.

## 5. Materials and Methods

### 5.1. Cell Lines

HEK293 cells (CRL-1573; American Type Culture Collection, Rochville, MD, USA) stably transfected with hOCT1 and hOCT3 were a kind gift of Prof. Koepsell (University of Würzburg) and grown with 600 µg/mL geneticin. HEK293 cells stably transfected with a hMATE1 plasmid (a gift by Dr. Yonezawa, Kyoto University Hospital, Japan Biochem. Pharmacol. 74: 359–371, 2007) were selected with 500 µg/mL hygromycin B. HEK293 cells transfected with cDNAs of hOCTN1 subcloned into a doxycycline-inducible pEBTetD plasmid vector were generously provided by Prof. Gründemann (University of Cologne, Cologne, Germany) [15,16] and selected with 3 mg/L puromycin. 24 h before starting experiments, hOCTN1 expression was induced by 1 mg/L doxycycline. 

All cells were grown under standard conditions in Dulbecco’s modified eagle medium (DMEM)—low glucose, containing 3.7 g/L NaHCO_3_, 1.0 g/L D-glucose, 10% foetal calf serum, 100 U/mL penicillin/streptomycin, 1 mM L-glutamine, gassed with 5% CO_2_ at 37 °C.

### 5.2. Synovial Fibroblasts (SF) Culture and Isolation

SF were isolated from synovial tissue of rheumatoid arthritis (RA) (*n* = 10) and osteoarthritis (OA) (*n* = 10) patients undergoing joint replacement surgery. The Ethics Committee of the University of Magdeburg approved this study (IRR: 73/18), and all patients gave written consent prior to inclusion in the study. RA patients met the American College of Rheumatology criteria. Isolated fibroblasts were cultured under standard conditions for maximal eight passages. When indicated, RA synovial fibroblasts (RASF) were incubated with 10 ng/mL recombinant human IL-6 (R&D). 

### 5.3. Peripheral Blood Mononuclear Cell (PBMC) Isolation and Cultivation

Human PBMCs were isolated from RA and OA patients. The Ethics Committee of the University of Magdeburg approved this study (IRR: 73/18) and all patients gave written consent prior to inclusion in the study. In brief, 10 mL blood samples were centrifuged at 400× *g* in a Megafuge (Thermofisher Scientific, Berlin, Germany) for 10 min at room temperature. The cell pellet was resuspended in PBS/0.1% BSA and centrifuged at 300× *g* at room temperature for 25 min without breaks using a Biocoll separating solution (Biochrom, Berlin, Germany). The generated lymphocyte ring was carefully taken off removed and washed two times twice with PBS/0.1% BSA. Cells were cultured in Roswell Park Memorial Institute medium (RPMI 1640, Sigma-Aldrich, Taufkirchen, Germany) supplemented with 2 mM l-glutamine, 10% foetal bovine serum, 1% penicillin/streptomycin solution at 37 °C with 5% CO_2_. 

### 5.4. Apparent Affinities of Baricitinib and Tofacitinib for OCTs With 4-(4-(Dimethylamino)styryl)-N-Methylpyridinium (ASP^+^)

To investigate whether Baricitinib and Tofacitinib interact with OCTs (hOCT1, hOCT3, hOCTN1, hMATE1, hMATE2k), we used a dynamic cis-inhibition protocol of ASP^+^ uptake, a known fluorescent substrate of all examined OCTs, with Baricitinib (10^−8^ to 10^−4^ M) and Tofacitinib (10^−8^ to 10^−4^ M), as described previously [14]. Briefly, HEK293 cells were seeded in 96-well plates and grown to 80–100% confluence. For experiments with hMATE-expressing cells, cellular pH was made acidic by 20 min preincubation with a modified ringer-like solution containing NH_4_Cl (in mM: 30 NH_4_Cl, 115 NaCl, pH 7.4). Baricitinib and Tofacitinib (Hycultech, Beutelsbach, Germany) were dissolved in DMSO in ringer-like solution (RLS = HCO_3_^−^ free Ringer-like solution containing (in mmol/L): NaCl 145, K_2_HPO_4_ 1.6, KH_2_PO_4_ 0.4, d-glucose 5, MgCl_2_ 1, calcium gluconate 1.3 with pH adjusted to 7.4) to final concentrations in the range 10^−4^–10^−9^ M. Fluorescence measurements were carried out with the TECAN® infinite F200 (Maennedorf, Switzerland). Transporter function was investigated measuring the slope of fluorescence emission (measured at 590 nm after excitation at 450 nm) increase in the first 60 s after ASP^+^ addition. ASP^+^ uptake without potential inhibitor was set to 100%. 

### 5.5. Quantification of Baricitinib and Tofacitinib Uptake by Liquid Chromatography Mass Spectrometry (LC/MS)

HEK293 cells or RASF/OASF seeded into a six-well plate were grown to 80–100% confluence. Medium was removed and cells were incubated with Baricitinib and Tofacitinib in RLS at 37 °C or 4 °C for 10 min. After this incubation, cells were quickly washed with 1 mL ice-cold RLS, and then lysed with 300 µL 0.1% formic acid. Cell lysates were incubated for 15 min in an ultrasound bath at 4 °C and centrifuged at 4700× *g* for 5 min at 4 °C. 10 µL internal standard (IS) of Baricitinib (Baricitinib-D5, Illkirch Graffenstaden, France) and Tofacitinib (Tofacitinib 13C3, Clearsynth, Mumbai, India) were added to the cell lysates for quantification. Acetonitrile (VWR, Radnor, Pennsylvania,, USA) was added, mixed and centrifuged for 15 min with 16,200× *g* at 4 °C. 200 µL of the supernatant were diluted in distilled water to reach a final acetonitrile concentration of 12%. The samples were frozen at −80 °C.

Quantification of Baricitinib and Tofacitinib concentration was performed using a high power liquid chromatography device (HPLC) (AdvanceTM UHPLC, Bruker Daltonik, Bremen, Germany) linked to a triple quadrupole mass spectrometer (EVOQ^®^ Elite triple quadrupole mass spectrometer, Bruker Daltonik, Bremen, Germany). A PAL HTC-xt autosampler (CTC Analytics AG, Zwingen, Switzerland) was used to inject 10 µl of the sample to the HPLC. Analytes were separated on an AccucoreTM C18 HPLC column (50 mm × 3 mm; 2.6 µm) (Thermo Scientific, Waltham, MA, USA). Amounts of 0.1% formic acid and acetonitrile were used as mobile phase A and B.

Flow rate was set to 1 mL/min. Baricitinib and Tofacitinib had a specific retention time in HPLC. Ionization of the substances was realized using electrospray ionization (ESI) in a positive ionization mode. Spray voltage was set to 3500 V, conus temperature to 350 °C, gas flow to 60 AU, sample and cone temperature to 350 °C. Exhaust gases were removed. First, Tofacitinib and Tofacitinib-IS, then Baricitinib and Baricitinib-IS were measured for 75 ms each. Full scan was applied to determine specific fission products of Baricitinib and Tofacitinib. Flow rate of the Cole-Parmer 74,900 single-syringe infusion pump (Vernon Hills, Illinois, USA) was set to 10 µL/min, spray voltage to 4000 V, gas flow to 10 AU, gas flow of the nebulizer to 10 AU and temperature to 25 °C. Quantification took place in multiple reaction mode (MRM) (Table 1). Each sample was measured three times and the mean was calculated thereof.

**Figure 5:**
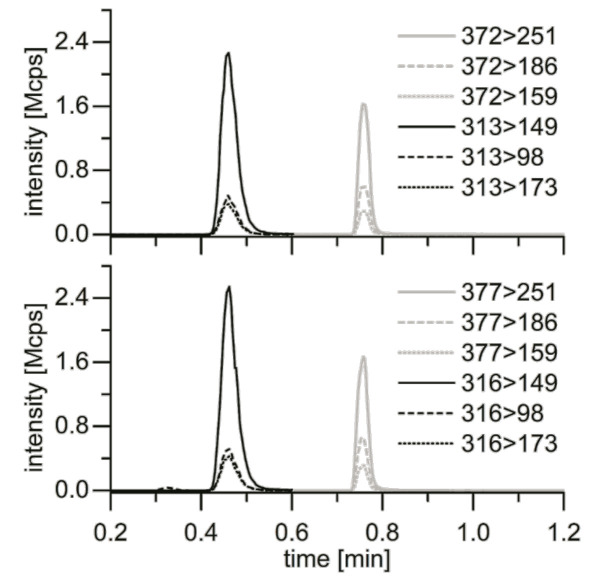
Transitions of Baricitinib and Tofacitinib (above) and IS (below). Tofacitinib is shown to the left in black, to the right Baricitinib in grey.

Quantification was attained comparing the content of Baricitinib and Tofacitinib in the sample to the added IS (Figure 5). The result was normalized to the protein content determined in Bradford assay. Analyses were performed using MS Workstation (Bruker Daltonik, Bremen, Germany), MS Data review Version 8.2 (Bruker Daltonik, Bremen, Germany), Origin Pro 2016 (OriginLab, Northampton, MA, USA), GraphPad Prism 5 (GraphPad Software, San Diego, CA, USA) and Excel 2016. Method optimization and validation resulted in detection limits of Baricitinib and Tofacitinib of 0.9 ng/mL and 1.0 ng/mL, and quantification limits of 3.0 ng/mL and 3.3 ng/mL, respectively.

### 5.6. Quantitative Real-Time PCR (qRT-PCR)

RNA was isolated with the Qiagen RNeasy Midikit (Qiagen, Gilden, Germany) and Invitrogen Super Script III system was used for reverse transcription. qRT-PCR was performed using SYBR Green PCR Master Mix and the ABI PRISM 7900 Sequence Detection System (Applied Biosystems, Darmstadt, Germany) (primer pairs see supplementary Table S1). Gene expression is normalized to a semiquantitative standard curve and given in relation to the housekeeping gene GAPDH.

### 5.7. Western Blot Analysis

For each RASF/OASF sample, a total of 1 × 10^6^ cells were seeded in a 25 cm^2^ cell culture flask and the cells became adherent overnight. The cells were stimulated with 10 ng/mL IL-6 for 0, 15, 30 and 60 min with either Tofacitinib (1 µM, 0.4 µM) or Baricitinib (1 µM, 0.15 µM). Cells lysis was performed using NP-40- buffer and proteinase inhibitor cOmplete Ultra. Cell lysates were run on a 10% SDS-PAGE and transferred to a PVDF membrane. Blocking was performed in 5% BSA solution. Primary antibody against phospho-Jak1 (#74129), Jak-1 (#3344), phosphor-Stat3 (#9145), Stat-3 (#30835), GAPDH (#2118) diluted 1:1000 in 5% BSA were incubated over night at 4 °C. The secondary antibody was an HRP-conjugated anti-rabbit (#7074) 1:8000 in 5% BSA. All antibodies were bought from Cell Signaling (Danvers, MA, USA). Enhanced chemiluminescence (ECL) was used for Western blot detection, and quantification of bands was performed using ImageJ Software (U.S. National Institutes of Health, Bethesda, MD, USA, https://imagej.nih.gov/ij/, 1997-201). 

### 5.8. Statistical Analysis

Data were analysed using GraphPad Prism, Version 5.0 (GraphPad Software, Inc., San Diego, CA, USA). To examine a statistical significance a two-way RM-ANOVA was performed. Sidak post-hoc multiple comparison test was performed to show intra individual significances. A *p*-value *p* ≤ 0.05 was considered to show statistical significance. All experiments were repeated independently for at least three times. 

## Figures and Tables

**Table 1:** MRM transitions of Baricitinib, Tofacitinib, IS and collision energy. The grey-marked fragments had the highest measurement intensity and were used for quantification.

Analyte	Transition [m/z > m/z]	Collision Energy
Tofacitinib	313 > 149	28
	313 > 98	31
	313 > 173	37
Tofacitinib-13C3	316 > 149	28
	316 > 98	31
	316 > 173	37
Baricitinib	372 > 251	26
	372 > 186	31
	372 > 159	43
Baricitinib-d5	377 > 251	26
	377 > 186	31
	377 > 159	43

## Supplementary Materials

Supplementary materials can be found at https://www.mdpi.com/1422-0067/21/18/6632/s1. Figure S1: Expression of organic cation transporters in PBMCs of OA and RA patients. Table S1: Primer for qRT-PCR.

## Abbreviations

ANOVA	Analysis of variance
ASP	4-(4-(dimethylamino)styryl)-*N*-methylpyridinium
AU	Arbitrary units
BSA	Bovine serum albumin
cDNA	complementary Deoxyribonucleic Acid
csDMARD-IR	conventional synthetic disease-modifying antirheumatic drug inadequate response
DMEM	Dulbecco’s modified eagle medium
DMSO	Dimethyl sulfoxide
ECL	Enhanced chemiluminescence
EMA	European Medicines Agency
ESI	electrospray ionization
GAPDH	Glyceraldehyde 3-phosphate dehydrogenase
gp130	Glycoprotein 130
HEK 293 cells	Human embryonic kidney 293 cells
HPLC	High Performance Liquid Chromatography
IL-6	Interleukin-6
IL-6Ra	Interleukin 6 Receptor Alpha
IS	Internal standrad
JAK	Janus Kinase
LC/MS	Liquid chromatography/mass spectrometry
MATE	multidrug and toxin extrusion
MRM	multiple reaction mode
MTX	methotrexate
NP-40	Nonidet P-40
OA	osteoarthrits
OC	Organic cation
OCT	Organic cation transporter
OCTN	Novel organic cation transporter
PBMCs	peripheral blood mononuclear cell
pKa	Ionization Constant
RA	Rheumatoid arthritis
RLS	ringer-like solution
RM-ANOVA	Repeated Measures Analysis of variance
RNA	Ribonucleic acid
RT-PCR	Reverse transcription polymerase chain reaction
SF	Synovial fibroblasts
STAT	Signal transducer and activator of transcription
TKI	Tyrosine kinase inhibitor
TNF-alpha	tumour necrosis factor alpha
TYK	Tyrosine kinase
