# Supplementary material for: Tofacitinib and Baricitinib Are Taken up by Different Uptake Mechanisms Determining the Efficacy of Both Drugs in RA
Source: Int J Mol Sci. 2020 Sep 10;21(18):6632. doi: 10.3390/ijms21186632 (PMC7556031; doi:10.3390/ijms21186632)
Supplement: Supplementary file 1 [file ijms-21-06632-s001.pdf]

Supplementary Materials

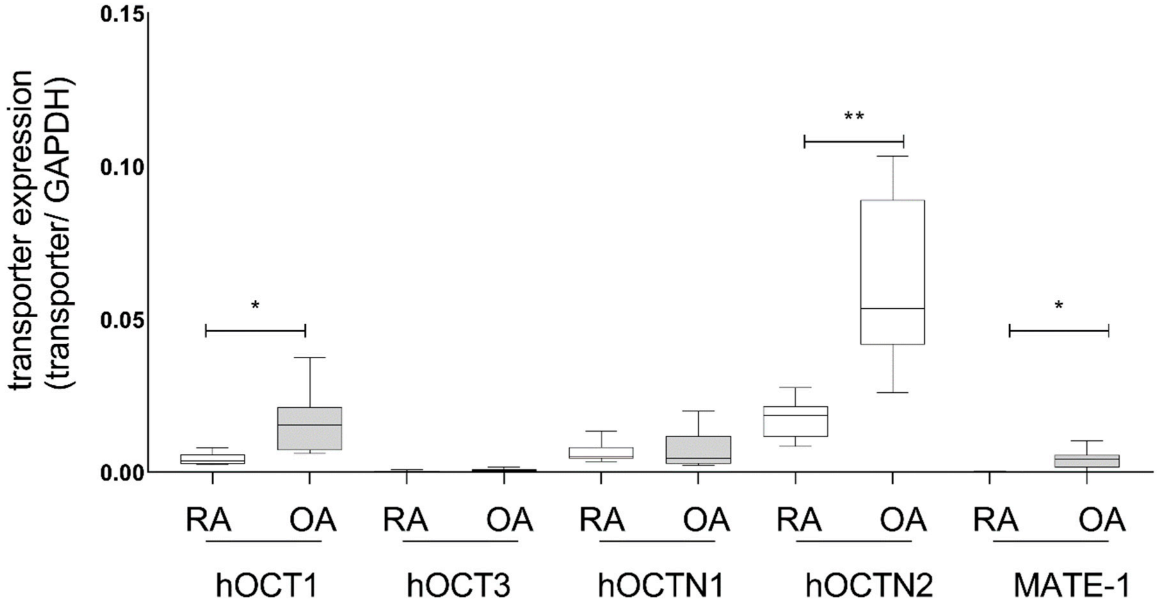

Supplementary Figure S1: Expression of organic cation transporters in PBMCs of OA and RA patients

Supplementary Table S1: Primer for qRT-PCR

Gen name		Primer sequence (5' → 3')
GAPDH	S	CAA GCT CAT TTC CTG GTA TGA C
	AS	GTG TGG TGG GGG ACT GAG TGT GG
OCT1	S	CAT CAT AAT CAT GTG TGT TGG CC
	AS	CAA ACA AAA TGA GGG GCA AGG CTT
OCT3	S	CCATCGTCAGCGAGTTTGAC
	AS	AAGTTTGGTGCAAAGGCCAC
OCTN1	S	TC CAG AAA CCT TAG AGC AGA TGC
	AS	GA ATG CAG TTA TTA GAA CCT TGG G
OCTN2	S	GTA CCC CAC TCC CAG ACA CC
	AS	GCTGTGCTTTTAAGGATTGTGGG
MATE-1	S	GCA ACC ACA CTT GGA GTG ATG G
	AS	GAG CAG AAT TCC CAC TCC GAG
S: Sense; AS: Antisense		
